# Appendagitis following Diagnostic Laparoscopy and Laparoscopic Appendicectomy

**DOI:** 10.1155/2016/6973061

**Published:** 2016-07-04

**Authors:** R. Kumar, R. F. Bamford, D. Kumar, G. Singh-Ranger

**Affiliations:** ^1^Department of Surgery, William Harvey Hospital, Ashford, Kent TN24 0LZ, UK; ^2^Department of Colorectal Surgery, St. George's Hospital and Medical School, Blackshaw Road, Tooting, London SW17 0QT, UK; ^3^Upper River Valley Hospital, Waterville, NB, Canada E7P 0A4

## Abstract

Appendagitis is an uncommon clinical entity, often not recognised, and mistaken for more serious infective conditions. We describe a proven case of appendagitis which occurred after confirmed appendicitis. We postulate that this condition can coexist with appendicitis and indeed may be the result of coinflammation. This has several implications. Firstly, clinicians must retain an index of suspicion for this condition in a patient with localised abdominal pain which occurs after appendicitis. Secondly, it would be reasonable to suggest careful examination of colocated appendages in a patient with an otherwise normal-appearing appendix. Treatment might require laparoscopic resection, as performed in this case.

## 1. Introduction and Case Report

A 21-year-old female presented to our emergency department with a history of right iliac fossa pain, loss of appetite, and nausea.

She had originally presented to a different hospital three weeks previously with the same symptoms and had been admitted. A laparoscopy had been performed, and her appendix vermiformis was removed. Subsequent histology revealed the appendix had been chronically inflamed.

She informed us that neither the operation nor the course of antibiotics prescribed postoperatively had made any difference. The rest of her history was unremarkable.

On examination, she had tenderness in the right iliac region, with guarding and percussion peritonism. Her temperature, blood tests, and urinalysis were normal.

**Figure 1:**
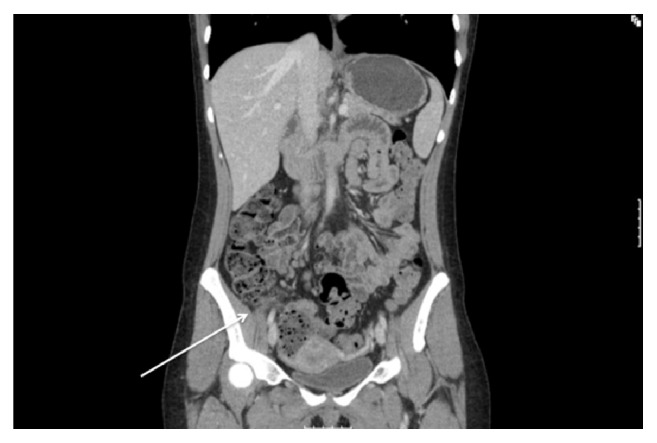
CT Scan of abdomen, supine view, demonstrating subtle loss of tissue plane between the caecal pole and surrounding areas.

She was admitted and initially managed conservatively. Ultrasound scans of her abdomen and pelvic organs were organised. These were normal, as were repeat blood tests after 24 hours. Gynaecological review was unremarkable. Despite analgesia, she continued to complain of pain and remained tender on examination. A CT scan of her abdomen and pelvis was organised as she was not improved and revealed inflammatory changes around the caecum, with enlarged mesenteric lymph nodes (Figure 1). There was no evidence of stump appendicitis on CT. A diagnostic laparoscopy was performed and revealed an inflamed appendix epiploica of the caecum. There were signs of a previous appendicectomy, a small ligated noninflamed appendix stump. Her uterus, ovaries, and tubes were normal. The inflamed appendage was removed and sent for histology. The patient's symptoms completely resolved after surgery, and she was discharged uneventfully. Subsequent histology confirmed necrosis and inflammation of the appendix epiploica.

## 2. Discussion

Epiploic appendages are pedunculated fatty structures on the external surface of the colon, arranged longitudinally in line with the taenia coli. They can extend from the caecum to the sigmoid colon and are covered by peritoneum and number around 50–100. They tend to spare the rectum [1–3].

Inflammation of these epiploicae, sometimes called “appendagitis,” is uncommon and can occur due to torsion of the epiploica or its vascular occlusion by thrombosis (primary appendagitis) or as a secondary phenomenon caused by the presence of inflammation nearby, for example, from sigmoid diverticulitis, cholecystitis, or pancreatitis [4, 5].

Most cases are probably due to primary causes, such as torsion, or vascular occlusion, and usually occur in the sigmoid colon [4, 5]; Singh et al. also found that it was the sole cause of the clinical symptoms in around 7% of cases of suspected diverticulitis.

With the advent of CT scanning in cases of acute abdominal pain, the diagnosis of appendagitis has been increasing. A recent CT review study estimated the frequency of epiploic appendagitis to be around 1.3%, with incidence of 8.8/million/year [6]. The condition typically presents in middle aged individuals, with a paucity of laboratory abnormality.

Our case is unique as appendagitis occurred after chronic appendicitis. This presentation probably occurred due to thrombosis of the appendage vessels caused by the chronically inflamed appendix. We postulate that although the appendix was removed, the appendicitis had a coproximal inflammatory effect which then manifested as appendagitis. Symptoms only resolved once the inflamed and necrotic appendage was removed at the patient's second laparoscopy.

It is possible that the patient may have had evolving appendagitis at the initial laparoscopy, but this may have been missed as attention would most likely have instead been paid to the appendix as being the source of the patient's problem. This emphasises the point that clinicians should have an index of suspicion for the appendage being abnormal.

As far as we are aware, there have been no other cases of appendagitis after appendicectomy reported in the literature.

A previous case report has described acute appendicitis occurring in a patient one week after laparoscopic treatment of acute appendagitis [7]. The appendix at the original procedure had been visualised as being macroscopically normal, which is why it was left alone. It is possible that the appendix was in fact inflamed at the time of the original laparoscopy and that the presence of the inflamed epiploica was simply a manifestation of “microscopic appendicitis.” There are histological signs of inflammation in up to 33% of “normal” appendicectomies [8]. With this in mind, we believe that the presence of appendagitis on imaging or at laparoscopy should be regarded as a sentinel for the presence of inflammatory disease in adjacent colon. This condition also forms a further rationale for removing the otherwise “normal-looking appendix” in a patient undergoing laparoscopy for suspected appendicitis in the absence of any other obvious cause for pain.

This case report reaffirms the need to consider the diagnosis of appendagitis in patients who present to emergency physicians with a history of right iliac region pain and a previous appendicectomy, especially in the absence of significant blood test abnormalities. Clinicians also need to have a low index of suspicion for inflammation elsewhere in the colon if appendagitis is found, particularly at laparoscopy, and the appendix vermiformis should be removed if caecal appendagitis is present, as highlighted by Chouillard et al.
